# *LTA*, *LEP*, and *TNF-a* Gene Polymorphisms are Associated with Susceptibility and Overall Survival of Diffuse Large B-Cell lymphoma in an Arab Population: A Case-Control Study

**DOI:** 10.31557/APJCP.2020.21.9.2783

**Published:** 2020-09

**Authors:** Sohaib M Al-Khatib, Nour Abdo, Laith N AL-Eitan, Abdel-Hameed W Al-Mistarehi, Deeb Jamil Zahran, Tariq Zuheir Kewan

**Affiliations:** 1 *Department of Pathology and Laboratory Medicine Jordan University of Science and Technology Irbid, Jordan. *; 2 *Department of Public Health, Faculty of Medicine, Jordan University of Science and Technology, Irbid, Jordan. *; 3 *Department of Biotechnology and Genetic Engineering, Faculty of Science and Arts, Jordan University of Science and Technology, Irbid, Jordan. *; 4 *Department of Family Medicine, Faculty of Medicine, Jordan University of Science and Technology, Irbid, Jordan. *; 5 *Department of Internal Medicine, Faculty of Medicine, Jordan University of Science and Technology, Irbid, Jordan.*; 6 *Department of Internal Medicine, Cleveland Clinic Foundation, Cleveland, Ohio, USA. *

**Keywords:** Diffuse large B-cell lymphoma, single nucleotide polymorphism, Arab population

## Abstract

**Objective:**

In this study, we aimed to explore the relationship between five selected proinflammatory and immune-mediated genes (TNF rs1800629G>A, rs361525G>A, rs1799964T>C, LTA rs1800683G>A, rs909253A>G, TNFAIP8 rs1042541C>T, LEPR rs1327118G>C, and LEP rs2167270G>A) and the risk and overall survival of DLBCL patients within the Jordanian Arab population.

**Methods:**

One hundred twenty-five patients (125) diagnosed with DLBCL at the King Abdullah University Hospital (KAUH) between 2013 and 2018 and 238 healthy cancer-free control subjects with similar geographic and ethnic backgrounds to the patients were included in the study. Genomic DNA was extracted from the formalin-fixed paraffin-embedded tissues of the subjects and from peripheral blood samples of the controls. The Sequenom MassARRAY^®^ sequencer system (iPLEX GOLD) was used. The analyses included assessments of population variability and survival.

**Results:**

Our study showed significant differences in the distribution of the studied polymorphisms of DLBCL between the patients and controls for TNF rs1800629G>A, LTA rs909253 G>A and LEP rs2167270 G>A. TNF rs1800629G>A (p = 0.01), in which the G allele harbors a higher risk of DLBCL (GG and GA genotypes when compared with AA genotype) (p = 0.044). The LTA rs909253 A>G polymorphism is associated with a higher risk of DLBCL in the allelic model (p = .004). LEP rs2167270 G>A polymorphism is associated with a decreased risk of DLBCL in the recessive mode models (p = .03). Subjects with the dominant model for TNF-a rs1799964 (TT genotype in comparison with the combined TT/TC genotype) and patients with the homozygous genotype (GG) of rs361525 have better overall survival rates.

**Conclusion:**

Our results confirmed the diversity and the heterogeneity of the disease. Although the study has a limitation because of its relatively small size, it clearly emphasizes the significance of ancestry and genetic composition as the determinants of DLBCL risk and behavior.

## Introduction

The World Health Organization’s (WHO) classification of hematolymphoid malignancies depends on a combination of morphologic, immunophenotypic, cytogenetic, and molecular findings.(Swerdlow et al., 2016) Accordingly, mature B-cell lymphomas are divided into Hodgkin and non-Hodgkin lymphoma types (HL and NHL). NHL is one of the most common hematologic malignancies with over 60 subtypes sharing the cell of origin but ranging in behavior from indolent to aggressive and very aggressive types. NHL is considered the sixth most common type of cancer and the ninth leading cause of cancer deaths among both males and females worldwide (Siegel et al., 2016; Teras et al., 2016). 

The incidence of non-Hodgkin lymphoma (NHL) is increasing, and in the United States alone, approximately 81,080 new cases have been diagnosed in 2016. That comprises 89% of all new lymphoma cases. However, the trends of NHL incidence are variable and show significant age, gender, racial, and geographic differences (Kamangar et al., 2006; Horesh and Horowitz, 2014; Ferlay et al., 2015; Perry et al., 2016; Batista et al., 2017). Generally, the overall incidence of NHL is higher in males, but females show greater extra-nodal involvement (Castillo et al., 2014). 

Regarding geographic variation, developing countries show lower incidence but higher grade of B-cell lymphomas in comparison with developed countries (Perryet al., 2016). The surrounding environment, infectious microorganisms, and lifestyle all play an important role in NHL pathogenesis (Morton et al., 2008; Crump et al., 2014; Ollberding et al., 2014; Slager et al., 2014).

Diffuse large B-cell lymphoma is the most common NHL (Harris et al., 2000). DLBCL is a diverse disease and can be classified into two genetically distinct types: the germinal center B-like type, characterized by the expression of germinal center B-cell genes, and the activated B-like type, characterized by the gene expression of activated peripheral blood B-cells. Clinically, the germinal center B-like type shows better overall survival (Alizadeh et al., 2000). The standard chemotherapy regimen for patients with DLBCL is the CHOP regimen (cyclophosphamide, doxorubicin, vincristine, and prednisone). Adding Rituximab (an anti-CD20 monoclonal antibody) increases the rate of complete remission and overall survival in comparison with the CHOP regimen alone (Coiffier et al., 2002). 

Several studies reveal that different genetic loci are linked with risk and/or outcome in DLBCL. Among them, the most notable genes are the IL6, IL10, LEPR, CTLA-4, IL4RA, TNF-α, and LT-a immunity genes (Berglund et al., 2005; Wang et al., 2006; Zhang et al., 2012). There is strong proof that changes in immunological functions pose an increased risk of lymphoma. Inflammatory and immune-response genes are the inventive messengers of adaptive immunity; they regulate the function of the immune system and growth of the lymphoid tissues.

In this study, we aimed to investigate the relationship between eight single nucleotide polymorphisms (SNPs) in five selected proinflammatory and immune-mediated genes (TNF rs1800629G>A, rs361525G>A, rs1799964T>C, LTA rs1800683G>A, rs909253A>G, TNFAIP8 rs1042541C>T, LEPR rs1327118G>C, and LEP rs2167270G>A) and the risk and overall survival of DLBCL patients in the Jordanian Arab population.

## Materials and Methods

*Patients and data collection*

One hundred twenty-five (125) patients and two hundred and thirty-eight (238) healthy cancer-free control subjects with similar environmental and racial backgrounds to the patients were included in the study. All cases of DLBCL diagnosed from the beginning of 2013 to the end of 2018 were collected from the archives of pathology of King Abdullah University Hospital. All cases were reviewed by (SK) and one representative section was chosen from each case. All the procedures in the study was approved by the Scientific Research Committee and Institutional Review Board (IRB) at the Jordan University of Science and Technology [ IRB code number 5/106/2017, dated 8/06/2017] in accordance with the 1964 Declaration of Helsinki and its later amendments. For the patients; the need for formal written informed consent was waived by the IRB. All control subjects were voluntarily involved and signed written informed consent. Cases’ and controls’ names were coded and blinded and treated confidentially.

*DNA analysis*

Formalin-fixed paraffin-embedded tissues of the DLBCL patients were used to extract the genomic DNA using the commercially available DNeasy Blood and Tissue Kit (Qiagen Ltd., West Sussex, UK), according to the manufacturer’s protocols. Peripheral blood samples were used to extract the genomic DNA of control healthy subjects using the QIAamp® or Promega DNA Mini Kit, in accordance with the manufacturer’s instructions. The quality of the extracted DNA was examined using agarose gel electrophoresis and ethidium bromide staining. The concentration and purity of the extracted DNA were assessed using a NanoDrop 1000^®^ spectrophotometer. The pure DNA samples and their concentrations were sent to the Australian Genome Research Facility (AGRF, Melbourne Node, Melbourne, Australia) for the genotyping of TNF (rs1800629G>A, rs361525G>A, and rs1799964T>C), LTA (rs1800683G>A and rs909253A>G), TNFAIP8 (rs1042541C>T), LEPR (rs1327118G>C), and LEP (rs2167270G>A). Genotyping with the Sequenom MassARRAY® system (iPLEX GOLD) (Sequenom, San Diego, CA, USA) was performed at the AGRF, as per the manufacturer’s recommendations (Sequenom, San Diego, CA, USA). The SNPs, SNP positions, and primer sequences for the TNF-a, LTA, TNFAIP8, LEPR, and LEP genes are shown in Table 1.

*Statistical analysis*

The follow-up period was calculated by subtracting the date of diagnosis from the date of death for the dead cases and by subtracting the date of diagnosis from the last updated date for the vital status of those patients who were alive until the last date of check-up. The actuarial life table survival analysis was used to obtain the overall survival probabilities. Cox’s proportional hazard regression was used to identify the independent factors related to survival. The survival analyses (genotypic, allelic, and clinical data association) were performed using the Statistical Package for the Social Sciences (SPSS), version 25.0 (SPSS, Inc., Chicago, IL). The survival curves were displayed using the GraphPad Prism 6 software. The SNPStats Web Tool was used to analyze the distribution of genotypes in the patients and the associations between polymorphisms and clinical variables (independent variables), including gender, age, and response to treatment. The patients’ clinical characteristics and response rates were compared using Chi square tests. The Hardy–Weinberg equilibrium was estimated through a goodness-of-fit *χ2* test. All the results were considered significant (P < 0.05). 

## Results

*Demographic and Clinical Data*

Table 2 summarizes patients’ demographic and clinical data. One hundred and twenty-five DLBCL patients and 238 ethnically and geographically matched healthy controls were enrolled in this study. Of the patients, 66 were male and 59 were female (52.8%/47.2%) with a mean age of 53.7 years. The mean age for the controls was 43.2 years (6–89) and 38.7% consisted of male subjects. Most of the patients (74%/59.2%) at the time of diagnosis were at the Ann Arbor stage IV of lymphoma. The mean levels for LDH, total protein, and serum albumin at the first encounter were 635 U/L, 58.6 g/dl, and 35.4 g/dl, respectively. The mean percentage of monocytes in the peripheral blood was 6.9%.

*Association between TNF, LTA, TNFAIP8, LEPR and LEP genes polymorphisms and the risk of DLBCL*

Eight single nucleotide polymorphisms (SNPs) in four inflammation and immune-related genes (TNF rs1800629G>A, rs361525G>A, rs1799964T>C, LTA rs1800683G>A, rs909253C>T, TNFAIP8 rs1042541C>T, LEPR rs1327118C>G, and LEP rs2167270G>C) were genotyped in all the subjects (patients and controls). 

The genotype distributions of the patients and controls were compared. Unconditional logistic regression analysis was used to estimate the association between the genotype frequency and the risk of developing DLBCL. Table 3 shows the distribution of both the genotypic and allelic frequencies of the eight SNPs. The allelic distribution did not reveal any significant differences in the distributions of the studied DLBCL polymorphisms between patients and controls except for TNF rs1800629 (p = o. o16) and LTA rs909253 (p = o.oo4). In our population, the patients with the G allele of TNF rs1800629 and A allele of LTA rs909253 had a higher risk of developing DLBCL in comparison with those having the A allele for TNF rs1800629 and the G allele for LTA rs909253.

Additional analyses based on four genetic models (codominant, dominant, recessive, and overdominant) shows that LEP rs2167270 G>A polymorphism is associated with a decreased risk of DLBCL in the recessive mode models (p = 0.03). The results are shown in Table 4.

*Association between TNF, LTA, TNFAIP8, LEPR and LEP genes polymorphisms and the survival rate of DLBCL*

**Figure 1:**
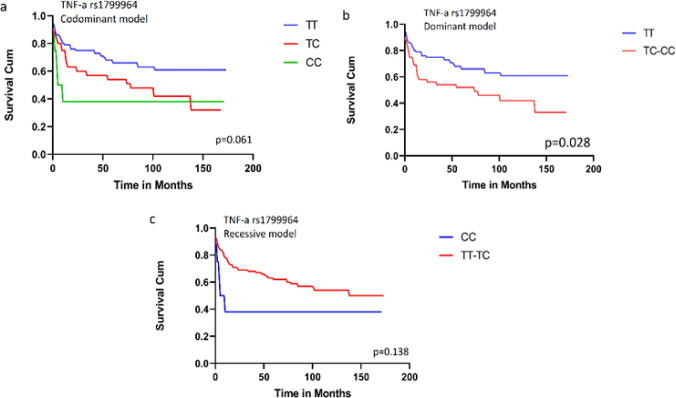
Overall Survival for 125 DLBCL Patients According to Genotype of TNF-a rs1799964. (a) Codominant model: comparison among TT, TC and CC genotypes; codominant model (p=.061). (b) Dominant model: comparison between TT genotype and combined TC/CC genotypes (p=0.028). (c) Recessive Model: comparison between CC genotype and combined TC/CC genotypes (p=0.138). Log-rank p values were indicated

**Figure 2:**
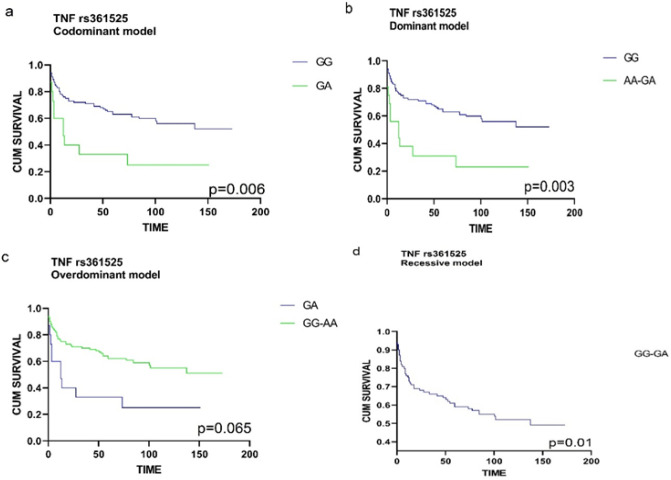
Overall Survival for 125 DLBCL Patients According to Genotype of TNF rs361525. (a) Codominant model: comparison among GG and GA genotypes (p=.006). (b) Dominant model: comparison between GG genotype and combined AA-AG genotypes (p=0.003). (c) Overdominant model: Comparison among GA genotype and combined GG-GA genotypes (p=0.01). (d) Recessive model: GG-GA genotype (p=0.065). Log-rank p values were indicated

All the 125 DLBCL patients were included in the survival analysis. Survival analyses were performed using the Kaplan-Meier curve and log-rank test. The only SNPs to show significant survival results were TNF-a rs1799964 and TNF rs361525. In our study, subjects with the dominant model for TNF-a rs1799964 (TT genotype in comparison with the combined TT/TC genotype) and patients with the homozygous genotype (GG) of rs361525 have better overall survival- rates. Figure 1 and Figure 2. 

## Discussion

Lymphomagenesis is a complex process involving an interaction between the tumor cells and the surrounding stromal cells or extracellular matrix (Balkwill and Mantovani, 2001). The tumor-microenvironment interaction is influenced by proinflammatory and immune-related factors that affect the proliferation and maturation of the interacting cells (Kurzrock, 1997). There is increasing evidence that the genetic composition of the host (gene polymorphism) influences incidence variation and the outcomes of patients with a similar tumor stage and histological grade (Bagg, 2004; Wang et al., 2009; Dunleavy et al., 2014; Glass et al., 2016). 

Based on their ability to kill mouse fibrosarcoma L-929 cells, two cytotoxic factors were subsequently reported: the first was the lymphotoxin (LT) factor, which is produced by lymphocytes and was recognized by Gale A Garner in 1968 (Kolb and Granger, 1968). And the second was the tumor necrosis factor (TNF), discovered in 1975 by LIoyd J. Old (Nedwin et al., 1985). The last exon is similar in both LT and TNF. Due to sequential homology in addition to functional homology, TNF was renamed as TNF-alpha, and LT was renamed as TNF-beta, they both belong to the TNF superfamily (Carswell et al., 1975). 

TNF is a proinflammatory cytokine secreted mainly by macrophages and involved in the regulation of cell proliferation, differentiation, and programmed cell death (Carswell et al., 1975; Männel et al., 1980; Nedwin et al., 1985). It also affects lipid metabolism, coagulation, and endothelial functioning (Pennica et al., 1984).

Tumor necrosis factor-alpha (TNF-a) is an acute phase reactant cytokine produced by many cells, including granulocytes, T-lymphocytes, and neurons, but mainly by macrophages. Its gene is located within the major histocompatibility complex (MHC) region of chromosome 6 (6p21.33), with biased expressions in the bone marrow and lymph nodes. As a functional protein, TNF-a has two forms: bound (integral/transmembrane) and soluble. The soluble form is released from its original integral membrane or transmembrane (TP) form by a cleavage enzyme (metalloprotease) called (ADAm17) (Black et al., 1997).

As an immune regulator, TNF-a is able to induce fever, cachexia, apoptosis, and inflammation. Additionally, it inhibits tumor genesis and viral replication. TNF binds to TNFR1, which is expressed by most cells, and activated by both the membrane-bound and soluble forms of TNF-a. However, TNFR2, which is primarily found in the immune system cells, is activated only by the membrane-bound form of TNF-a (Theiss et al., 2005).

Protein signaling in the TNF–TNFR1 interaction occurs through the tumor necrosis factor receptor type 1-associated DEATH domain protein (TRADD), a 34 kDa adaptor protein that interacts with an intracellular domain (death domain) of TNFR1 after its dissociation from the inhibitory protein, SODD. The overexpression of TRADD is responsible for two major roles of TNF: anti-apoptosis and inflammation by activating the NF-ΚB pathway (Hsu et al., 1995). The inflammatory response of TNF is also induced by the inflammatory, pro-apoptotic MAPK pathway. The FADD/Caspase 8 pathway plays a minor role in the TNF induction of cell death (Chen and Goeddel, 2002; Lavrik et al., 2005).

TNF is produced by the TNF gene cluster, which consists of three functional genes (TNFA, TNFB, and LTB) (Browning et al., 1993). Sequencing the entire coding region of the TNF-a gene shows the presence of five single nucleotide polymorphisms (SNP)—four in the upstream region and one in the non-translated region. In the upstream region, and with respect to the TNF transcriptional start site, there are three SNPs characterized by the substitution of adenine for guanine at nucleotides -238 (rs361525), -308 (rs1800629), and -376 (rs1800750) (Herrmann et al., 1998; Knight et al., 1999). The first two SNPs (rs361525 and rs1800629) are found in the promoter region, and they lead to increased TNF expression. These SNPs have been implicated in a variety of diseases, including autoimmune diseases, diabetes, coronary heart diseases, and cancer (Norman et al., 1995; Herrmann et al., 1998; Zinman et al., 1999). Additionally, the level of TNF-a is associated with poor prognosis and can expect treatment outcome in lymphoma patients (Salles et al., 1996). Among the most widely studied polymorphisms in TNF-a is -308G/A (rs1800629), in which the A allele is associated with an increase in transcriptional activity and the level of TNF-alpha. Reported studies from different institutions and geographic regions show no consistent relationships between -308G/A polymorphism and the risk of DLBCL. Both positive and negative associations have been reported between subjects of the same ethnicity and/or country of origin. For example, among American and Norway Caucasians, both Morton et al., (2008) and Yri et al., (2013) reported an increased risk of DLBCL with TNF -308G/A polymorphism (Morton et al., 2008; Yri et al., 2013). But Skibola et al., (2010) and Thunberg et al., (2010) found no such associations (Skibola et al., 2010; Thunberg et al., 2010). Among Asians, the results are inconsistent; some studies indicate that TNF-a G308A polymorphism is significantly associated with an increased risk of DLBCL while others show negative associations (Xiao and Zhang, 2011; Hosgood et al., 2013; Zhai et al., 2014). Our study among the Jordanian Arab population showed a positive association and an increased risk of DLBCL associated with TNF rs1800629G>A in the co-dominant, dominant, and recessive models [odds ratio 2.18, 2.19, and 2.16, respectively; 95% confidence interval (CI) 1.11–4.31, 1.14–4.22, and 1.10–4.25, respectively; p value = 0.047, 0.014, and 0.016, respectively]. The conflicting results of different studies indicate that the genetic effect of TNF-a 308G/A polymorphism on NHL risk is ethnically and geographically dependent and may be partially explained by different allele frequencies among different populations (6%, 5%, 7%, and 13% in Jordanian, South Asian, American, and European populations, respectively).

Among our notable findings, the rs1799964T>C SNP in the TNF-a gene was associated with better overall survival (OS) in DLBCL patients. Kaplan-Meier analyses of overall survival rates revealed that patients with a dominant genotype (TT genotype in comparison with combined TC/CC genotypes) of TNF rs1799964 had a higher overall survival rate (log-rank p = 0.028).

Lymphotoxin-A (LTA), or tumor necrosis factor beta (TNF-b), acts as a modulator in the immune system; it is a key regulator in lipid metabolism, secreted by lymphocytes, and has 35% identity and 50% homology in the amino acid sequence of TNF-alpha (Gray et al., 1984; Lo et al., 2007). The genetic location of LTA is (6p21.33) and induces its functions through the TNF alpha receptor-1 (TNFRSF1A/TNFR1) and TNF beta receptor-2 (TNFRSF1B / TNFR2) (Aggarwal et al., 1985).

We found that lymphotoxin-a (LTA) 252A>G (rs909253) polymorphism was associated with an increased risk of DLBCL among our population (p = o.oo4), a result that is in concordance with previous reports in studies among the non-Hispanic white populations (Rothman et al., 2006).

The tumor necrosis factor-alpha-induced protein 8 (TNFAIP8) is a protein with 188 amino acids and a molecular mass of 21 kD, located on chromosome 5q23.1 (Horrevoets et al., 1999). TNFAIP8 is an early anti-inflammatory cytokine and apoptosis regulator that negatively regulates T-cell receptor signaling, is involved in oncogenesis, and is found in many normal tissues and malignant cells. Its expression is induced by TNF-alpha through NF-KB pathway activation (Kumar et al., 2000). Head and neck squamous cell carcinoma, esophageal squamous cell carcinoma, breast cancer, and non-small cell lung carcinoma show an increased expression of TNFAIP8 mRNA (Patel et al., 1997; Dong et al., 2010; Hadisaputri et al., 2012). However; the association between TNFAIP8 (rs1045241C>T) polymorphism and NHL, DLBCL in particular, is not well explored. In a study by Yan et al., (2012), the presence of TNFAIP8 (rs1045241C>T) polymorphism was significantly associated with the overall risk of DLBCL and follicular lymphoma (FL) among a Chinese population (Zhang et al., 2012). In our study, no significant relationship was found between TNFAIP8 rs1045241 C>T polymorphism and the risk of DLBCL.

Leptin, or the obese (OB) gene, is a 16-kD endocrine (adipocyte-derived) protein mapped to chromosome 7 (7q32.1) and has an important role in regulating appetite, basal body metabolism, body weight, insulin level, and inflammatory response (Matsuda et al., 1997; Tian et al., 2002). Leptin mediates its effects through a cytokine single transmembrane domain receptor called leptin receptor (LEPR). Accordingly, leptin and LEPR are associated with the pathogenesis of obesity, diabetes mellitus (DM), blood pressure, and cancers (Skibolaet al., 2004; Lin et al., 2015; Zhang et al., 2018). Leptin gene expression is increased by sugar-rich diets and reduced by fasting. Polymorphisms in leptin (OB) and LEPR affect body weight homeostasis in different ways (Coleman, 1978; Thompson et al., 1997). LEP rs2167270 G>A polymorphism, which involves the untranslated 5-prime region of the gene and results in a single A-to-G transition in the 26th nucleotide, is associated with low leptin levels and morbid obesity (Hager et al., 1998). The obese phenotype is also associated with LEPR rs1137101 polymorphism, which results in the replacement of glutamine by arginine at codon 223 (Q223R (Gotoda et al., 1997). However, the relationship between body mass index (BMI) and the risk of cancers, including hematologic malignancies, is not well established, and previous studies show inconsistent results (Franceschi, et al., 1989; Wolk et al., 2001; Cerhan et al., 2002). In their studies, Skibola et al., (2004) and Willett et al., (2005) found that the presence of A allele associated with LEP rs2167270 G>A polymorphism decreases the risk of NHL, but both LEP rs7799039 G>A and LEPR rs1137101 (Q223R) polymorphisms are associated with increased risk of NHL (Skibola et al., 2004; Willett et al., 2005). 

These findings contradict the results of an Asian Chinese population study, in which no relationship was found between LEP rs2167270G>A and LEPR rs1327118C>G polymorphisms and risk of NHL (Zhang et al., 2012). Our results showed no significant association between LEPR rs1327118 G>C polymorphism and the patient’s susceptibility to DLBCL. However, contrary to what was reported for the Chinese population, we found that LEP rs2167270 G>A polymorphism is associated with a decreased risk of DLBCL in the recessive mode models [odds ratio 0.47; 95% confidence interval (CI) 0.24–0.93; p = 0.032].

**Table 1:** The SNPs, SNPs Position and Primers Sequences for *TNF-a, LTA, TNFAIP8, LEPR*, and *LEP* Genes

SNP-ID	Gene	Chr^	bp*	Primer Forward	Primer Reverse
rs1800629	*TNF-a*	6	31543031	ACGTTGGATGCTGATTTGTGTGTAGGACCC	ACGTTGGATGGGAGGCAATAGGTTTTGAGG
rs361525	*TNF-a*	6	32145194	ACGTTGGATGAAGCATCAAGGATACCCCTC	ACGTTGGATGCACACAAATCAGTCAGTGGC
rs1799964	*TNF-a*	6	31542308	ACGTTGGATGCTACATGTGGCCATATCTCC	ACGTTGGATGGGGAAGCAAAGGAGAAGCTG
rs1800683	*LTA*	6	3154007	ACGTTGGATGTAGTCCAAAGCACGAAGCAC	ACGTTGGATGTCTATAAAGGGACCTGAGCG
rs909253	*LTA*	6	31540313	ACGTTGGATGAGAGAAACCCCAAGGTGAGC	ACGTTGGATGTCTCTGTCACACATTCTCTG
rs1042541	*TNFAIP8*	5	76221370	ACGTTGGATGGGGAGGTAAGGAAGGAAATT	ACGTTGGATGGTGATACTCACTCTCTACCC
rs1327118	*LEPR*	7	65885569	ACGTTGGATGAACACTGGTTAGTGAGTCGG	ACGTTGGATGTCCTGAGAACGGAGATGTAG
rs2167270	*LEP*	7	128241296	ACGTTGGATGTGTGGCCTGCCAAGAAAGAC	ACGTTGGATGATGGAGCCCCGTAGGAATC

* bp, base pair (Genomic Position); ^ Chr, Chromosome

**Table 2:** Demographic and Clinical Data of 125 Lymphoma Patients of Jordanian Arab Descent in This Study

Category	Value N(%)	
	Cases	Controls
Demographic Data		
Gender		
Male	66 (52.8)	92 (38.7)
Female	59 (47.2)	146 (61.3)
Age in Years*		
0-14	4 (3.2)	3 (1.3)
15-19	4 (3.2)	18 (7.6)
20-40	18 (14.4)	89 (37.4)
41-55	31 (24.8)	59 (24.8)
>55	68 (54.4)	69 (28.9)
Mean (Range)	53.7 (1 - 89)	43.2 (6 - 89)
Median (IQR)	57 (44 - 66)	44 (24.2 - 57)
Clinical Data		
Survival Status		
Alive	69 (55.2)	----
Dead	56 (44.8)	----
Survival Months		----
Median	55	----
B-Symptoms		
Yes	15 (14.9)	----
No	86 (85.1)	----
Ann Arbor Stage at Diagnosis		
0	2 (1.6)	----
1	25 (20)	----
2	9 (7.2)	----
3	11 (8.8)	----
4	74 (59.2)	----
Unknown	4 (3.2)	----
Serum LDH		
Mean (Range)	635 (2 - 4422)	----
Median (IQR)	423 (194.5 - 790)	----
Total Protein		----
Mean (Range)	58.6 (4 - 93.3)	----
Median (IQR)	65.8 (57 - 73)	----
Serum Albumin		----
Mean (Range)	35.4 (3 - 87.8)	----
Median (IQR)	38.4 (33 - 43)	----
Total Monocytes		
Mean (Range)	6.9 (1 - 22)	----
Median (IQR)	6.3 (4.3 - 8.5)	----

*Age of controls vs age at diagnosis in cases. Competition

**Table 3:** The Frequency of Allele and Genotype for Eight Proinflammatory and Immune Related SNPs in DLBCL Patients and Controls (Case / Control)

SNP ID	Cases N (%)	Controls N (%)	*P-*value
rs1800629			
Allele G	224 (94.0)^	418 (89.0)^	0.016
Allele A	14 (6.0)^	54 (11.0)^	
Genotype G/G	106 (89.1) ^	186 (78.8) ^	0.0443 **
Genotype G/A	12 (10.1) ^	46 (19.5) ^	
Genotype A/A	1 (0.8) ^	4 (1.7) ^	
rs361525			
Allele G	233 (93.0) ^	443 (93.0) ^	0.94
Allele A	17 (7.0)^	33 (7.0) ^	
Genotype A/A	1)1.0 (^	1)0.0 ( ^	0.94**
Genotype G/A	15)12.0 (^	31)13.0 ( ^	
Genotype G/G	109)87.0 (^	206)87.0 (^	
rs1799964			
Allele T	182 (76.0)^	378 (80.0)^	0.26
Allele C	56 (24.0)^	94 (20.0)^	
Genotype T/T	71 (59.7) ^	149 (63.1) ^	0.27
Genotype T/C	40 (33.6) ^	80 (33.9) ^	
Genotype C/C	8 (6.7) ^	7 (3.0) ^	
rs1800683			
Allele G	158 (72.0)^	309 (67.0)^	0.16
Allele A	60 (28.0)^	151 (33.0)^	
Genotype G/G	59 (54.1)	105 (45.6)	0.34
Genotype G/A	40 (36.7)	99 (43)	
Genotype A/A	10 (9.2)	26 (11.3)	
rs909253			
Allele A	163 (70.0)^	314 (68.0)^	0.004
Allele G	43 (30.0)^	146 (32.0)^	
Genotype A/A	59 (52.0) ^	109 (47.0) ^	0.58
Genotype A/G	45 (40.0) ^	96 (42.0) ^	
Genotype G/G	9 (10.0) ^	25 (11.0) ^	
rs1045241			
Allele C	179 (79.0)^	324 (74.0)^	0.23
Allele T	49 (21.0)^	112 (26.0)^	
Genotype C/C	69 (61.0)^	123 (56.0)^	0.3
Genotype C/T	41 (36.0)^	78 (36.0)^	
Genotype T/T	4 (4.0)^	17 (8.0)^	
rs1327118			
Allele G	119 (52.0)^	251 (54.0)^	0.68
Allele C	109 (48.0)^	215 (46.0)^	
Genotype G/G	34 (29.8) ^	66 (28.3) ^	0.47
Genotype G/C	51 (44.7) ^	119 (51.1) ^	
Genotype C/C	29 (25.4) ^	48 (20.6) ^	
rs2167270			
Allele G	148 (63.0) ^	316 (69.0) ^	0.08
Allele A	88 (0.37) ^	140 (31.0) ^	
Genotype G/G	49 (42.0) ^	107 (47.0) ^	0.08
Genotype G/A	50 (42.0) ^	102 (45.0) ^	
Genotype A/A	19 (16.0) ^	19 (8.0) ^	

^, Allele frequency and percent. The allele frequency might be counted twice in each person. **, Fisher's Exact Test (33% of the cells have expected counts less than 5. Chi-Square may not be a valid test.)

**Table 4:** Genotype Distribution of the TNF, LTA, LEPR, TNFAIP8, and LEP SNPs in DLBCL Patients

SNP ID	Model	Genotype	OR^ (95% CI)	P-value
rs1800629 TNF-a	Codominant	G/G	1	0.047
		G/A	2.18 (1.11-4.31)	
		A/A	2.28 (0.25-20.66)	
	Dominant	G/G	1	0.014
		G/A-A/A	2.19 (1.14-4.22)	
	Recessive	G/G-G/A	1	0.019
		A/A	2.03 (0.22-18.41)	
	Overdominant	G/G-A/A	1	0.016
		G/A	2.16 (1.10-4.25)	
rs361525 TNF-a	Codominant	G/G	1	0.87
		G/A	1.09 (0.57-2.11)	
		A/A	0.53 (0.03-8.54)	
	Dominant	G/G	1	0.86
		G/A-A/A	1.06 (0.56-2.01)	
	Recessive	G/G-G/A	1	0.65
		A/A	0.52 (0.03-8.44)	
	Overdominant	G/G-A/A	1	0.78
		G/A	1.10 (0.57-2.12)	
rs1799964 TNF-a	Codominant	T/T	1	
		T/C	0.92 (0.56-1.50)	0.38
		C/C	0.46 (0.16-1.38)	
	Dominant	T/T	1	
		T/C-C/C	0.84 (0.52-1.35)	0.47
	Recessive	T/T-T/C	1	
		C/C	0.48 (0.16-1.40)	0.18
	Overdominant	T/T-C/C	1	
		T/C	0.97 (0.59-1.57)	0.89
rs1800683 LTA	Codominant	G/G	1	
		C/G	0.91 (0.54-1.53)	0.89
		C/C	1.26 (0.24-6.66)	
	Dominant	G/G	1	0.79
		C/G-C/C	0.93 (0.56-1.54)	
	Recessive	G/G-C/G	1	
		C/C	1.30 (0.25-6.78)	0.76
	Overdominant	G/G-C/C	1	
		C/G	0.91 (0.54-1.52)	0.71
rs909253 LTA	Codominant	A/A	1	
		G/A	1.15 (0.72-1.86)	0.58
		G/G	1.50 (0.66-3.43)	
	Dominant	A/A	1	
		G/A-G/G	1.21 (0.77-1.90)	0.4
	Recessive	A/A-G/A	1	
		G/G	1.41 (0.63-3.13)	0.39
	Overdominant	A/A-G/G	1	
		G/A	1.08 (0.68-1.71)	0.73
rs1042541 LEPR	Codominant	C/C	1	0.27
		C/T	1.07 (0.66-1.72)	
		T/T	2.38 (0.77-7.37)	
	Dominant	C/C	1	0.47
		C/T-T/T	1.18 (0.75-1.88)	
	Recessive	C/C-C/T	1	0.11
		T/T	2.33 (0.76-7.08)	
	Overdominant	C/C-T/T	1	0.97
		C/T	0.99 (0.62-1.59)	
		Genotype	OR^ (95% CI)	P-value
		G/G	1	0.48
		C/G	1.20 (0.71-2.04)	
		C/C	0.85 (0.46-1.58)	
		G/G	1	0.77
		C/G-C/C	1.08 (0.66-1.76)	
		G/G-C/G	1	0.31
		C/C	0.76 (0.45-1.29)	
		G/G-C/C	1	0.27
		C/G	1.29 (0.82-2.02	
		G/G	1	0.097
		G/A	0.93 (0.58-1.51)	
		A/A	0.46 (0.22-0.94)	
		G/G	1	0.34
		G/A-A/A	0.80 (0.51-1.26)	
		G/G-G/A	1	0.03
		A/A	0.47 (0.24-0.93)	
		G/G-A/A	1	0.67
		G/A	1.10 (0.70-1.72)	

^, Odd Ratio
